# Structural insights into human EMC and its interaction with VDAC

**DOI:** 10.18632/aging.205660

**Published:** 2024-03-15

**Authors:** Mingyue Li, Chunli Zhang, Yuntao Xu, Shaobai Li, Chenhui Huang, Jian Wu, Ming Lei

**Affiliations:** 1 Ninth People’s Hospital, Shanghai Jiao Tong University School of Medicine, Shanghai 200011, China; 2 Shanghai Institute of Precision Medicine, Shanghai 200125, China; 3 State Key Laboratory of Oncogenes and Related Genes, Shanghai Jiao Tong University School of Medicine, Shanghai 200025, China

**Keywords:** EMC, VDAC, membrane proteins biogenesis, gating plug, cryo-electron microscopy

## Abstract

The endoplasmic reticulum (ER) membrane protein complex (EMC) is a conserved, multi-subunit complex acting as an insertase at the ER membrane. Growing evidence shows that the EMC is also involved in stabilizing and trafficking membrane proteins. However, the structural basis and regulation of its multifunctionality remain elusive. Here, we report cryo-electron microscopy structures of human EMC in apo- and voltage-dependent anion channel (VDAC)-bound states at resolutions of 3.47 Å and 3.32 Å, respectively. We discovered a specific interaction between VDAC proteins and the EMC at mitochondria-ER contact sites, which is conserved from yeast to humans. Moreover, we identified a gating plug located inside the EMC hydrophilic vestibule, the substrate-binding pocket for client insertion. Conformation changes of this gating plug during the apo-to-VDAC-bound transition reveal that the EMC unlikely acts as an insertase in the VDAC1-bound state. Based on the data analysis, the gating plug may regulate EMC functions by modifying the hydrophilic vestibule in different states. Our discovery offers valuable insights into the structural basis of EMC's multifunctionality.

## INTRODUCTION

The endoplasmic reticulum (ER) plays a key role in maintaining cellular homeostasis. Cellular aging can elevate ER stress, resulting in protein folding problems that may lead to the accumulation of misfolded proteins linked to age-related diseases [1, 2]. The ER membrane protein complex (EMC) is a multiple-subunit complex that is highly expressed in the ER membrane and plays a central role in the biogenesis of membrane proteins [3–5]. The EMC has a broad range of client proteins, including tail-anchored proteins, N-terminal and multi-pass transmembrane proteins [6, 7]. Specifically, the EMC is responsible for the post-translational insertion of low-hydrophobic transmembrane helix (TMH) of TA proteins [7], as well as for the co-translational insertion of the first helix of some multi-pass transmembrane proteins into the lipid bilayer [6]. Beyond its function as an insertase, the EMC also acts as a chaperon during membrane protein folding, assembly and quality control [8–11], and is involved in diverse cellular processes such as virus replication [12, 13] and the ER-mitochondria crosstalk [14–16]. In accordance with its diverse clients, the human EMC has been associated with a variety of pathological phenotypes like cancer, type 2 diabetes and neurological disorders [17–22], underscoring its essential roles in human health.

Recent cryo-electron microscopy studies on human and yeast EMC have unveiled a conserved architecture of the complex with three modules respectively located in the ER lumen, membrane and cytosol [23–27]. The EMC contains two distinct transmembrane cavities - the hydrophilic vestibule and the lipid-filled hydrophobic groove - present on opposite sides of the complex. The hydrophilic vestibule, which is structurally similar to other members of the Oxa1 superfamily insertases, such as YidC, GET1, and TMCO1, penetrates about halfway through the membrane and acts as a conduit for substrate TMH-insertion [28–30]. The membrane-embedded vestibule provides enough room to accommodate a low-hydrophobic substrate-TMH, and contains conserved positively charged residues that are important for substrate insertion [26, 27]. In addition, it has been proposed that this substrate-TMHs cavity of EMC might be regulated through a transmembrane gating mechanism [23–26]. Yet, there is no structural evidence to support this hypothesis.

The voltage-dependent anion channel (VDAC), lying in the mitochondrial outer membrane, regulates metabolite exchange between mitochondria and other cellular compartments. VDAC-interacting protein complexes are formed under physiological and pathological conditions, regulating metabolic, apoptotic, and other processes that may be impaired in disease [31]. Here, we present cryo-electron microscopy structures of human EMC in apo and VDAC-bound states at resolutions of 3.47 Å and 3.32 Å, respectively. Comparison of these structures unveils a gating plug formed by a segment of EMC3, which substantially modifies the client-binding pocket in different states. We speculate that the VDAC-bound state of EMC may represent other roles for the complex, rather than an insertase. Our findings provide insights into the structural basis of the EMC and its multifunctionality, facilitating a better understanding of disease-related EMC phenotypes in the future.

## RESULTS

### The structure of apo human EMC

**Figure 1:**
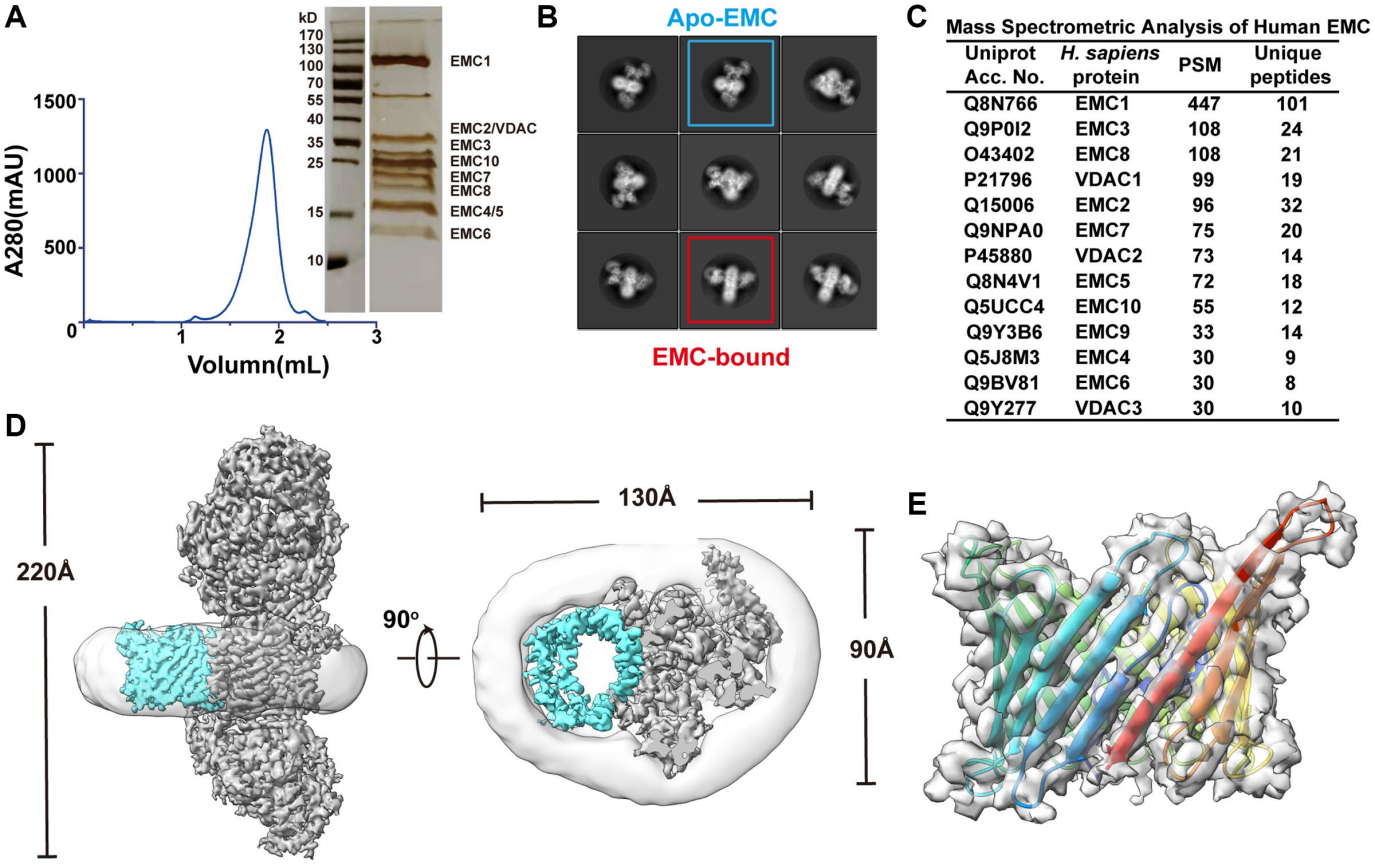
**Discovery and structural study of the VDAC1-bound EMC complex.** (**A**) Left panel: size-exclusion chromatography profile of the purified human EMC complex. Right panel: a silver staining image of the SDS-PAGE gel corresponding to the peak fraction in the profile. (**B**) Representative reference-free 2D cryo-EM average of the EMC. (**C**) Mass spectrometry analysis of the purified human EMC. (**D**) Two orthogonal views of the cryo-EM density map of human EMC-VDAC. The EMC, VDAC, and detergent micelle are colored in cyan, gray, and light gray respectively. (**E**) Side view of the cryo-EM density map and the ribbon diagram of human VDAC1, colored in rainbow to show the arrangement of the 19 β-strains.

**Figure 2:**
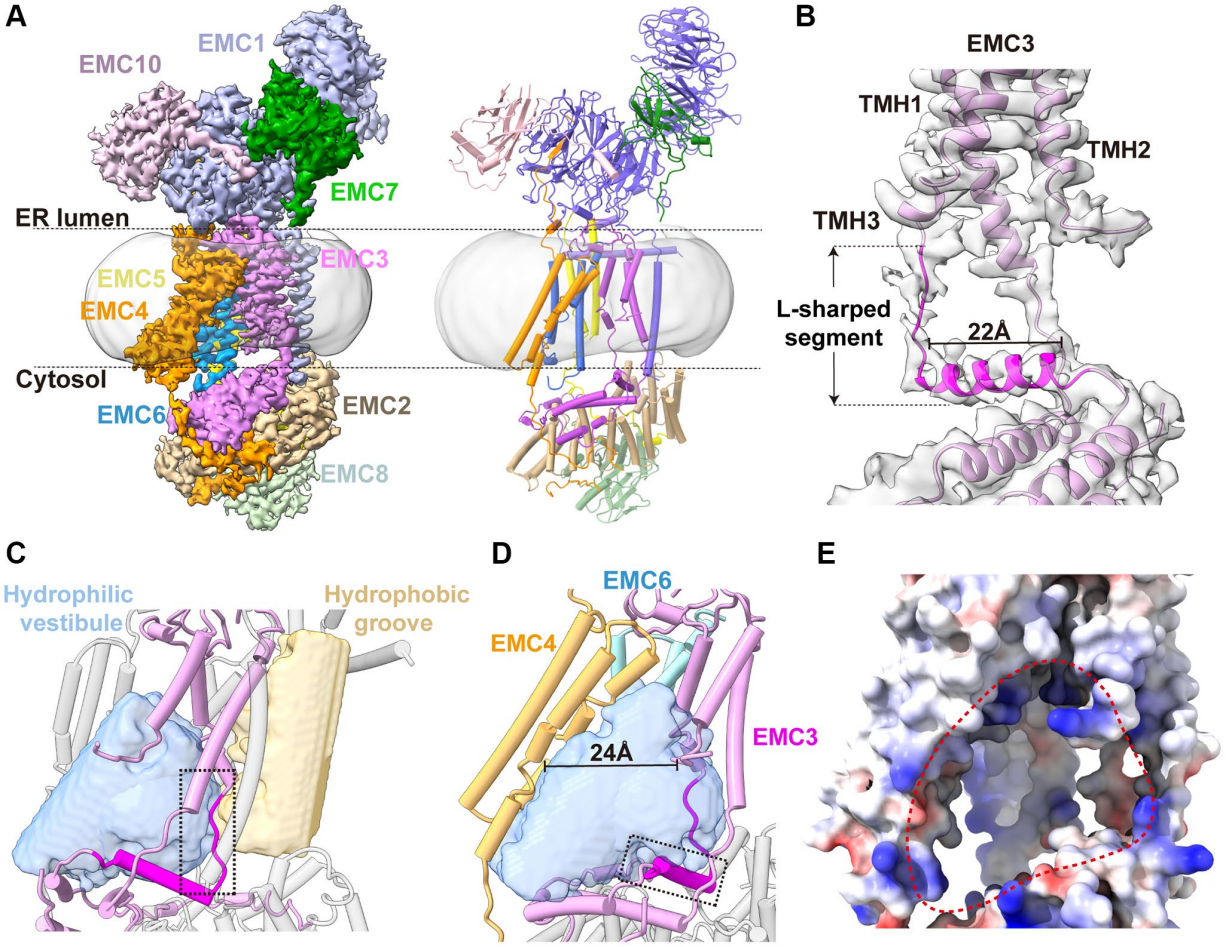
**Cryo-EM structure of human EMC.** (**A**) The cryo-EM density map and ribbon diagram of the human EMC complex, color-coded to highlight the subunit arrangement. (**B**) A close-up view of the EMC3 transmembrane module. The L-shape Segment^188–210^ of EMC3 are highlighted in magenta, and the rest of the EMC3 colored in plum. (**C**) A close-up view of the transmembrane module showing the two transmembrane cavities in the EMC. The internal cavities, determined by program HOLLOW, are shown as surface representation and the EMC in cylinder model. The extended loop of the L-shape Segment^188–210^, which separates the hydrophilic vestibule from the hydrophobic cavity, is highlighted by a dashed box. (**D**) The front view of the horseshoe-shaped hydrophilic vestibule. The L-shaped EMC3 segment and the nine-TMH bundle of EMC3-EMC4-EMC6 are shown. The horizontal helix of the L-shaped EMC3 segment that forms the base of the hydrophilic vestibule, is highlighted by a dashed box. (**E**) The front view of the EMC transmembrane region in electrostatic surface potential (positive: blue; negative: red). The entrance of the hydrophilic vestibule is defined by a dashed circular loop.

To obtain insights into the arrangement of the dynamic subunits of human EMC in the transmembrane module, we improved the homogeneity of the human EMC sample by purifying the endogenous EMC in digitonin from Expi293F cells. This was achieved using a two-step affinity purification scheme with a 3xFlag tag on EMC2 and a Twin-Strep tag on EMC5, followed by size exclusion chromatography (SEC) (Figure 1A). The highly purified human EMC sample was subjected to single-particle cryo-EM analysis. During data processing, reference-free two-dimensional class averages and three-dimensional classification unveiled multiple conformations of the transmembrane module (Figure 1B; Supplementary Figure 1), which prevented us from achieving a high-resolution human EMC structure. To address this issue, we applied various computational techniques including optimizing the box size of particles, selecting particles with maximum resolution of 4 Å, and utilizing no-alignment 3D classification. These efforts resulted in a well-defined class of apo human EMC with an overall resolution of 3.47 Å (Supplementary Figure 1). The resulting density map was of sufficient quality to enable us to build the atomic model of human EMC (Figure 2A; Supplementary Table 1).

The apo structure of human EMC exhibits a tripartite organization, of which the luminal and cytosolic modules are consistent with the recent structures of human EMC (Figure 2A; Supplementary Figure 2) [24–27]. In the transmembrane module, a three-TMH bundle of EMC4 adjacent to the EMC3-EMC6 core is ordered, forming the sidewall of the hydrophilic vestibule (Figure 2A, 2D). The most striking finding of our human EMC structure is the clear densities we observed for EMC3 residues 188–210 between TMH3 and the C-terminal helix, which are only partially visible in previous human EMC structures (Figure 2B) [27]. This L-shaped EMC3 segment starts with an extended loop (residues 188–193) that traverses the transmembrane module and separates the hydrophilic vestibule from the hydrophobic cavity (Figure 2C). Afterwards, the loop makes a sharp turn to fold into a 22-Å horizontal helix atop the cytosolic module, forming the base of the hydrophilic insertase vestibule (Figure 2D, 2E). It is noteworthy that the ‘loop-helix’ structural pattern of this EMC3 segment is conserved from yeast to humans as predicted by the Alpha-fold analysis, indicating its potential importance in the insertase function of EMC. (Supplementary Figure 3A).

Collectively, our human EMC structure unveils the complete architecture of the horseshoe-shaped insertase cavity of EMC, which is enclosed by the L-shaped EMC3 segment and the nine-TMH bundle of EMC3, EMC4 and EMC6 (Figure 2D). The insertase cavity opens laterally to the lipid environment of the ER membrane between EMC3 and EMC4, creating a ~24-Å entrance for the assess of substrate-TMH (Figure 2D; Supplementary Figure 3B). While members of the Oxa1 insertase superfamily share a basic common transmembrane fold [32], the size of the EMC insertase cavity is much larger than those in YidC and GET1-GET2 (Supplementary Figure 3B), suggesting that this conserved transmembrane scaffold has evolved to cope with different substrates for distinct functions.

### Structure of the human EMC-VDAC1 complex

During cryo-EM data processing we identified a second major class of particles that contains an extra β-barrel-type density adjacent to the transmembrane module of human EMC (Figure 1B; Supplementary Figure 1). To reveal the identity of this additional density, we examined the composition of human EMC using mass spectrometry analysis and found that three paralogous proteins of voltage-dependent anion channel 1–3 (VDAC1, VDAC2 and VDAC3) that adopt a typical β-barrel conformation were co-purified with the EMC (Figure 1C, 1D). Among these paralogs, VDAC1 was the most abundant one in the MS data, likely due to its widespread expression in mammalian cells [33, 34]. Docking of VDAC1 in the density map revealed that most interface residues on VDAC1 are conserved in the other two paralogs (Figure 1E; Supplementary Figure 4A). Hereafter, we will focus on the interaction between EMC and VDAC1 unless stated otherwise.

**Figure 3:**
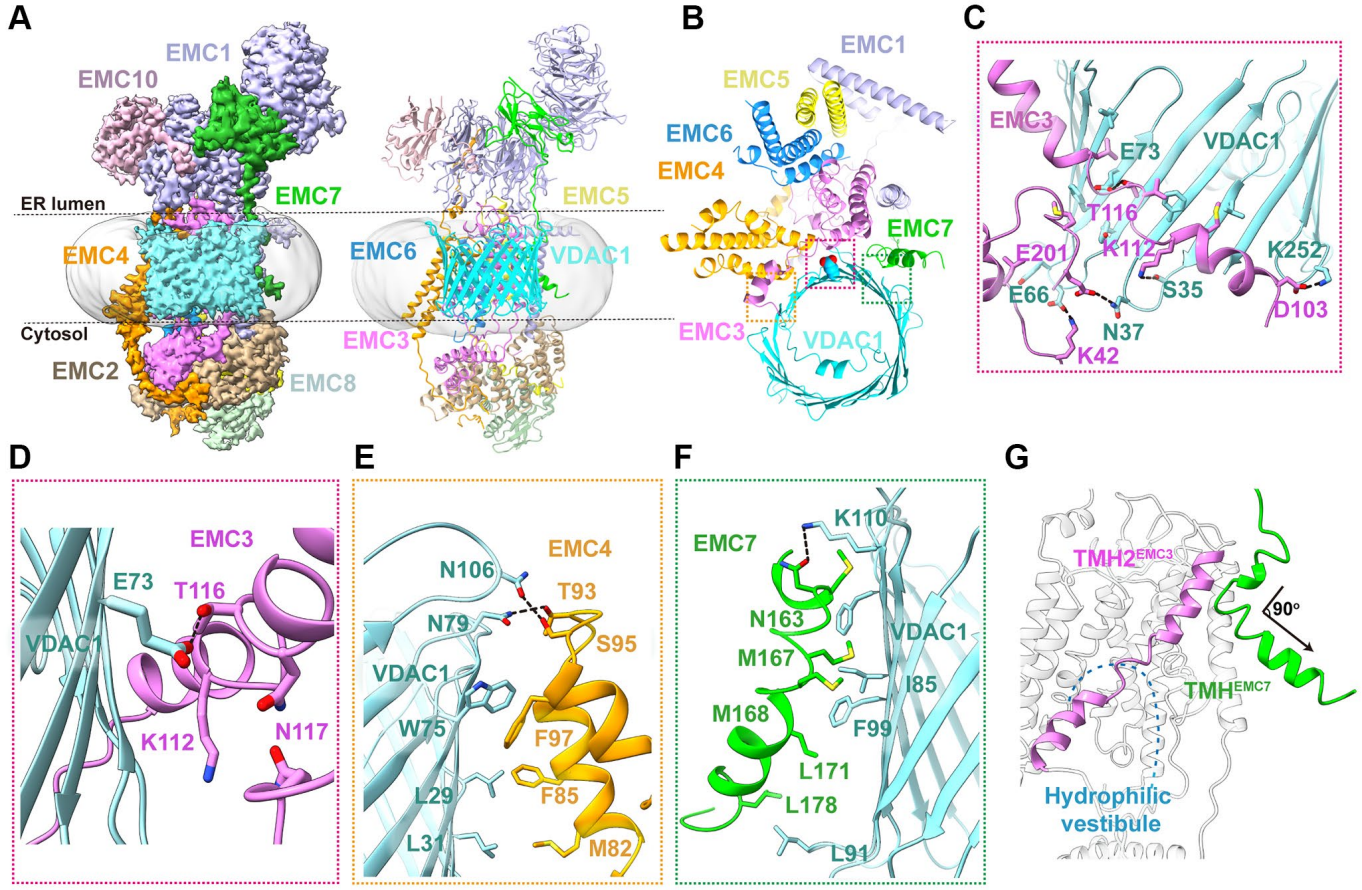
**Cryo-EM structure of human EMC-VDAC1 complex.** (**A**) The cryo-EM density map and ribbon diagram of human EMC-VDAC1 complex, color-coded by subunit. (**B**) The top view of the complex showing the interaction between VDAC1 and EMC. (**C**) A close-up view of the EMC3-VDAC1 interface highlighted in the pink dashed box in (**B**). Charged amino acids Lys42, Lys112, and Asp103 of EMC3 respectively interact with residues Glu66, Ser35, and Lys252 in the cytosolic loops of VDAC1. (**D**) Glu73^VDAC1^ is buried inside a hydrophilic pocket formed by Lys112, Thr116 and Gln117 of EMC3. (**E**) A zoom-in view of the EMC4-VDAC1 interface indicated by the orange dashed box in (**B**). The interaction involves hydrogen bonds formed by Asn79 and Asn106 of VDAC1 with Ser95 and Thr93 of EMC4, respectively. (**F**) The hydrophobic interface between EMC7 and VDAC1, mediated by Met167, Met168 and Leu178 of EMC7, and Ile85, Phe99 and Leu91 of VDAC1. (**G**) A close-up view of TMH^EMC7^. This segment adopts a bent conformation, with a short helix adjacent to TMH2^EMC3^ followed by another helix turning away from the hydrophilic vestibule.

The human EMC-VDAC1 complex has a 1:1 stoichiometry and measures a dimension of ~220 × 130 × 90 Å^3^ in size (Figures 1D, 3A). The transmembrane region is composed of 13 transmembrane helices from six EMC complex subunits (EMC1 and EMC3-EMC7) and a β-barrel pore of VDAC1 (Figure 3A, 3B). VDAC1 consists of 19 β strands, and the well-resolved N-terminal segment is positioned within the pore and aligned with the inner surface of the barrel wall (Figures 1E, 3B). Both the N- and C-termini of VDAC1 are located at the same side as the cytosolic subunits of human EMC (Figure 3A). Superposition analysis of EMC-bound VDAC1 structure with those of the ATP-bound mouse VDAC1 (PDB 4C69) [35] or NADH-bound human VDAC1 (PDB 6TIR) [36] unveiled a structural kinship between EMC-bound and ATP-bound VDAC1 structures (Supplementary Figure 4B), suggesting that EMC associated VDAC1 adopts an open conformation. This is in accordance with the observation that VDAC1 is predominantly in the open state in the absence of a membrane potential [37].

The EMC-VDAC1 interface involves extensive interactions between one side of the VDAC1 barrel and three EMC subunits EMC3, EMC4 and EMC7, burying an exposed surface area of ~2,336 Å^2^ (Figure 3B). First, the TMHs of EMC3 are positioned proximal to the outwall of VDAC1 mainly via hydrophobic contacts (Figure 3C). A panel of hydrogen bonds and salt bridges between EMC3 and the cytosolic loops of VDAC1 further buttress these hydrophobic interactions (Figure 3C). In particular, Glu73 of VDAC1 is buried inside a hydrophilic pocket formed by Lys112, Thr116 and Gln117 of EMC3 (Figure 3D). The second binding interface in the EMC-VDAC1 complex is between the TMH1 and TMH2 of EMC4 and the β1–β4 surface of VDAC1 through both hydrophobic and hydrophilic interactions (Figure 3E). A surprising finding of the human EMC-VDAC1 complex structure is that, unlike in the apo EMC structure, the TMH of EMC7 is ordered in the complex and make close contacts with strands β7–β8 of VDAC1 via several aliphatic and aromatic residues, contributing about one-third of the interface between EMC and VDAC1 (Figure 3F). TMH^EMC7^ adopts a bent conformation, with a short helix adjacent to TMH2^EMC3^ followed by another helix turning ~90° counterclockwise away from the hydrophilic vestibule (Figure 3G). Taken together, these structural observations suggest that human EMC could mediate an extensive interaction with VDAC1, and especially TMH^EMC7^ that is completely invisible in the apo EMC, plays an important role in the EMC-VDAC1 interaction.

### *In vivo* association between EMC and VDAC1

**Figure 4:**
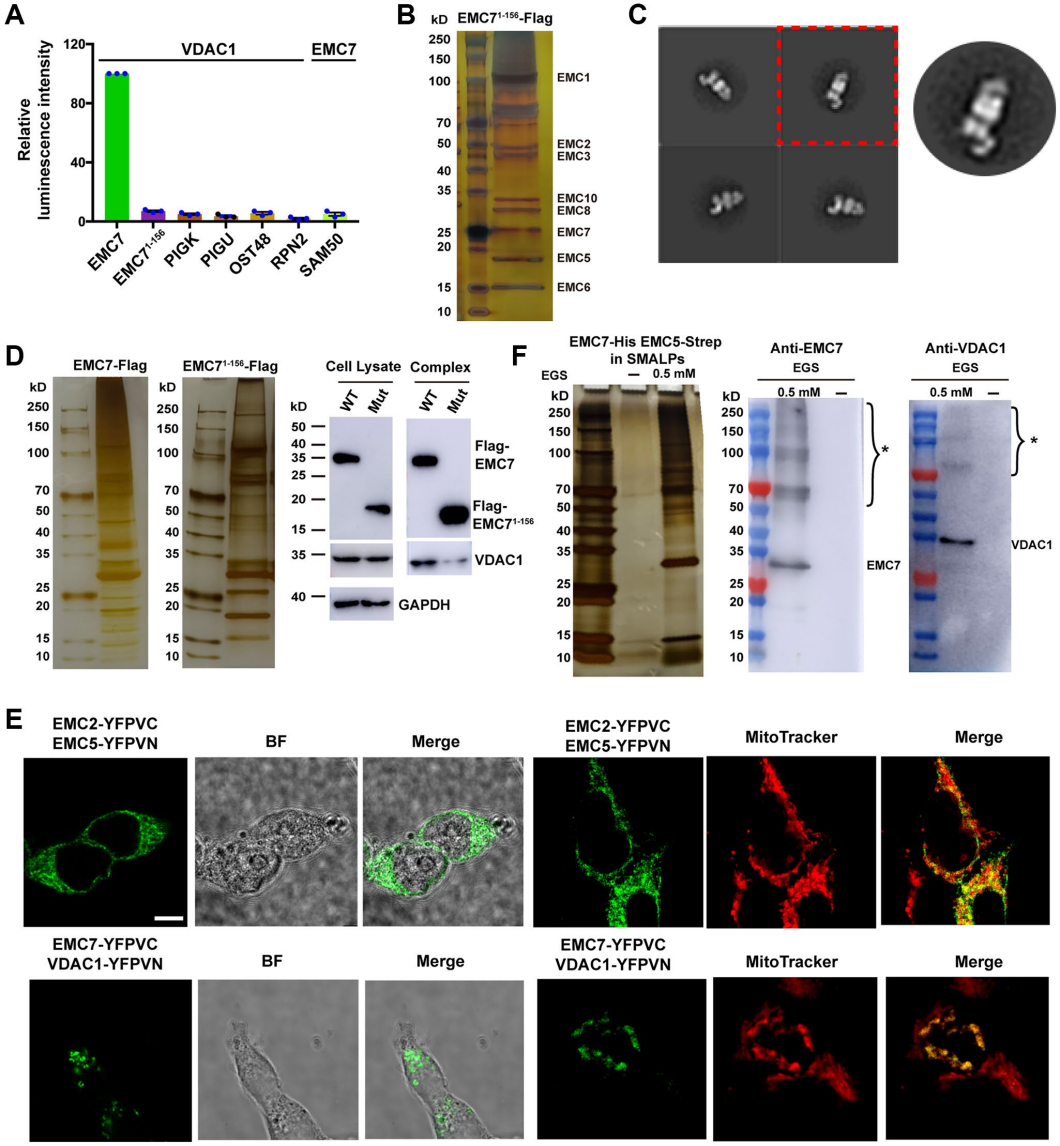
***In vivo* association between EMC and VDAC1.** (**A**) ER membrane proteins PIGK, PIGU, OST48 and RPN2 were tagged on their cytoplasmic side. Mitochondrial outer membrane protein SAM50 was tagged at its C-terminus. Luminescence intensities were measured to quantify the strength of the protein-protein interactions, and the intensity of VDAC1 with EMC7 was set as 100 for each experiment. The result represents mean ± s.e.m from *n* = 3 independent experiments. (**B**) A silver staining image of the SDS-PAGE gel for the human EMC complex purified from Expi293 cells that lack endogenous EMC7 but express C-terminal Flag-tag EMC7^1–156^ ectopically. (**C**) Representative reference-free 2D negative staining-EM average of the purified mutant EMC. (**D**) The truncation mutant EMC7^1–156^ attenuates the formation of EMC-VDAC1 complex. Left: Silver-stained SDS-PAGE of the purified EMC complex. Right: Western blot analysis of the purified EMC complex and whole cell lysate using antibodies as indicated. (**E**) BiFC assay examining the location of the EMC-VDAC1 complex. Proteins are fused either to the N-terminal fragment (-YFPVN) or to the C-terminal fragment (-YFPVC) of YFP. Mitochondria were stained by MitoTracker probes. BF: Bright field. Scale bar: 5 μm. (**F**) Purification of the EMC complex in the native lipid environment. Left: Silver-stained SDS-PAGE of the purified EMC complex in SMALPs with or without EGS crosslinker. Right: Western blot analysis of the purified EMC complex using antibodies as indicated. ^*^ indicates the cross-linked form of the target proteins.

Next, we set out to examine whether the EMC-VDAC1 association takes place *in vivo*. To address this issue, we utilized the NanoBiT proximity assay to analyze the interaction between EMC7 and VDAC1 in HEK293T cells [38]. We observed clear luminescence signals with the combination of C-terminally fused luciferase fragments with VDAC1 and EMC7, confirming that the association between EMC7 and VDAC1 indeed occurs in cells (Figure 4A). The mitochondrial outer membrane protein SAM50 and ER membrane proteins such as subunits of the GPI transamidase complex (PIGK and PIGU) were used as controls to validate the specificity of the EMC7-VDAC1 interaction (Figure 4A). These results indicate that the observed luminescence signal was specific for the interaction between VDAC1 and EMC7. Notably, the EMC7^1–156^ deletion mutant, which lacks the TMH that mediates the interaction with VDAC1 but has no effect on the EMC assembly (Figure 4B, 4C), exhibited no association with VDAC1 (Figure 4A). Moreover, complementation with EMC7^1–156^ deletion mutant in EMC7 knockout cells substantially impaired the formation of the EMC-VDAC1 complex (Figure 4D). The results demonstrate the vital role of TMH^EMC7^ in the interaction between EMC and VDAC1 *in vivo*.

We further employed the BiFC assay to investigate the location of the EMC-VDAC1 complex in living cells by expressing split-YFP fragments respectively fused with EMC7 and VDAC1 [39, 40]. Consistent with the NanoBiT measurement, the BiFC result revealed assembled YFP signals for the combination of VDAC1 and EMC7 (Figure 4E). Notably, the observed YFP signals were located at mitochondria-ER contact sites *in vivo* (Figure 4E). By contrast, when EMC2 and EMC5 were used in the assay, the assembled fluorescent signals appeared at the ER membrane (Figure 4E). These findings suggest that VDAC1 is specifically associated with EMC at mitochondria-ER contact sites. Moreover, by crosslinking with ethylene glycol bis (EGS), we found that the EMC-VDAC1 complex could be purified in the native lipid environment within styrene maleic acid lipid particles (SMALP) [41] (Figure 4F), suggesting that the EMC-VDAC1 interaction occurs *in situ* within cells.

Both EMC and VDAC1 are conserved from yeast to humans [4, 42]. We next explored whether the binding of VDAC1 to EMC is also conserved in yeast. By introducing a TAP tag on EMC7 and a 3xFlag tag on Por1 (yeast homologue of human VDAC1) in budding yeast *Saccharomyces cerevisiae*, we purified the yeast EMC-VDAC1 complex using a two-step affinity scheme (Supplementary Figure 5A). Mass spectrometry analysis confirmed the presence of VDAC1 and all core EMC components (yeast EMC1-EMC7 and EMC10) in the complex (Supplementary Figure 5B), suggesting that yeast Por1 could also binds to the EMC. Taken together, these results support that the formation of the EMC-VDAC1 complex is evolutionarily conserved from yeast to human.

### Structural changes between the apo EMC and EMC-VDAC1

**Figure 5:**
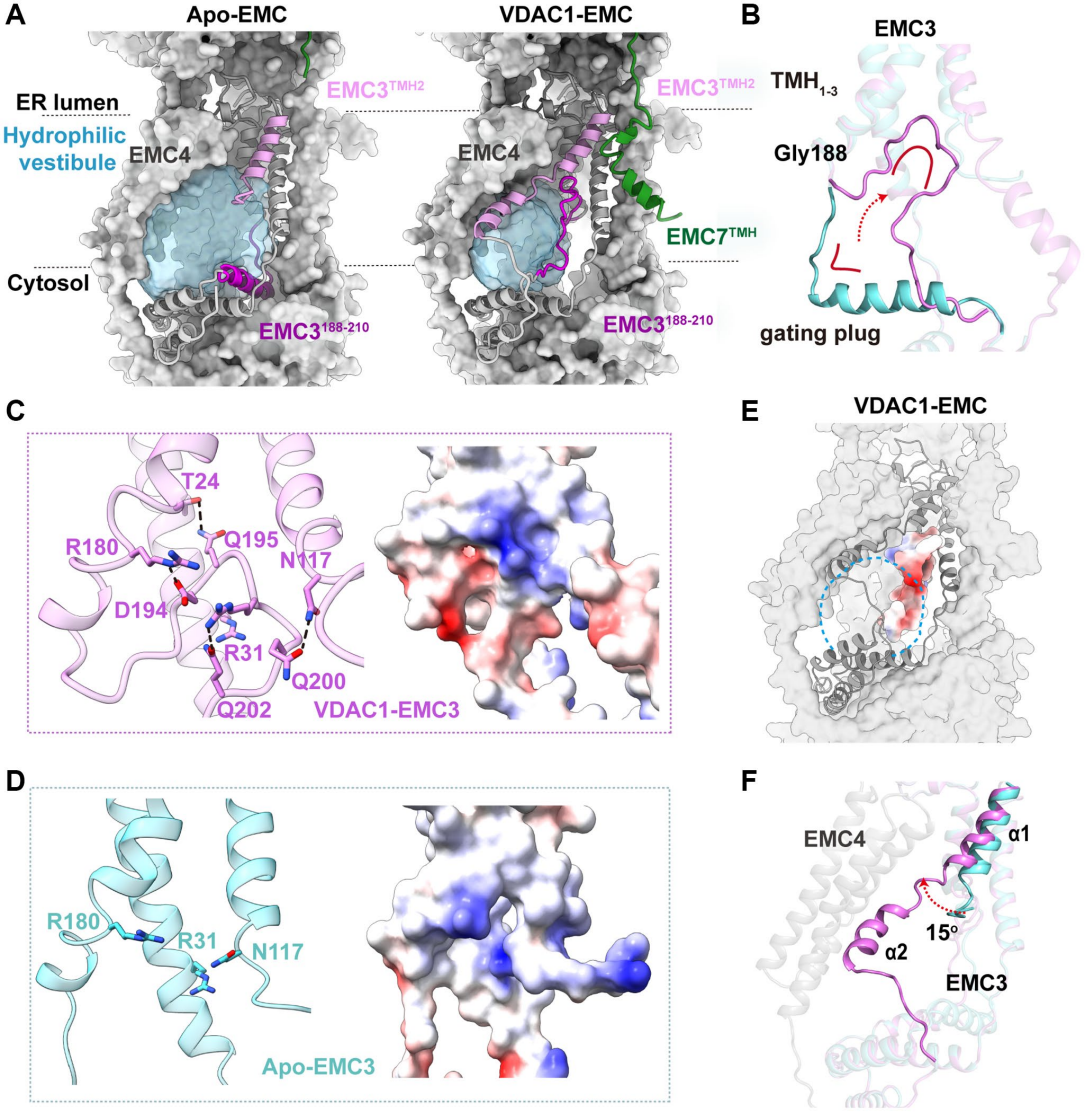
**Conformational changes during the transition between the apo- and VDAC1-bound EMC.** (**A**) Comparison of the hydrophilic cavities in the apo and VDAC1-bound EMC. The cavities are shown as surface representation in light blue, and EMC3 and EMC7 in ribbon, with TMH^EMC7^, TMH2^EMC3^ and EMC3^188–210^ segment colored in green, orchid and dark orchid, respectively. (**B**) Conformational change of EMC3^188–210^ segment, the gating plug, between the apo and VDAC1-bound states. This segment forms an L-shape architecture (cyan) in the apo EMC state, whereas it folds back around Gly188 to form an open circular loop (orchid), held against the TMH_1–3_ bundle of EMC3 in the VDAC1-bound state. (**C**) Left: the intramolecular interactions of EMC3 that stabilize the gating plug in the VDAC1-bound state. Hydrogen bonds are indicated by dashed lines. Right: electrostatic surface potential of the same region. (**D**) Residues Arg31, Arg180 and Asn117 on the TMH_1–3_ bundle of EMC3 form a conserved positively charged patch inside the EMC hydrophilic vestibule in the apo state. (**E**) Electrostatic surface potential of the EMC3 gating plug. The location of hydrophilic cavity is indicated by a dashed circle. (**F**) Conformational change of TMH2^EMC3^ between the apo and VDAC1-bound states. In the apo EMC (cyan), TMH2^EMC3^ is only partially ordered (α1). In the VDAC1-bound state (orchid), the entire TMH2^EMC3^ (α1 and α2) is visible.

The atomic models of the EMC in both apo and VDAC-bound states provide us with a unique opportunity to gain insights into conformational changes in human EMC during the transition between different states. The most striking observation is that the EMC3^188–210^ segment undergoes a dramatic transformation upon VDAC1 binding (Figure 5A, 5B). This segment forms an L-shape architecture in the apo state, playing an important role in the formation of the hydrophilic vestibule (Figure 5B). In stark contrast, it folds back around a hinge residue Gly188 to form an open circular loop, which is tightly held against the TMH_1–3_ bundle of EMC3 in the VDAC1-bound state (Figure 5B). The tethering of this circular loop is stabilized by extensive hydrogen-bond and salt-bridge interactions with adjacent TMH_1–3_ of EMC3 (Figure 5C). This segment may function as a ‘gating plug’ and regulate the shape and the surface electrostatic property of hydrophilic vestibule in the EMC. Upon VDAC1 binding, a conserved positively charged patch on TMH_1–3_ of EMC3 inside the EMC hydrophilic vestibule is completely occluded by the negative charges from the gating plug after its translocation (Figure 5C, 5D) [43]. Furthermore, the dramatic conformational change of the gating plug also substantially alters the inside shape of the hydrophilic vestibule, and greatly reduces the available space for potential substrate accommodation (Figure 5A, 5E).

Besides the EMC3 gating plug, the TMH2 of EMC3 also exhibits clear structural changes during the apo-to-VDAC-bound transition (Figure 5F). In the apo state, only a portion of the TMH2 in EMC3 is visible, which protrudes away from the hydrophilic vestibule (Figure 5F). By contrast, the entire TMH2 of EMC3 is ordered in the EMC-VDAC1 complex, consisting of two helices α1 and α2 joined by a short loop (Figure 5F). Helix α1 rotates by ~15° towards the direction to the TMHs of EMC4, and α2 traverses along the TMHs of EMC4, leading to the closure of the entrance to the hydrophilic vestibule (Figure 5A). Similarly, TMH^EMC7^, which is also highly flexible and thus invisible in the apo EMC structure, adopts a bent configuration and forms an ordered TMH closely adjacent to the TMH2 of EMC3 in the EMC-VDAC1 complex (Figure 5A). It is likely that the structural ordering of TMH^EMC7^ is induced by the extensive interactions between TMH^EMC7^ and VDAC1. Taken together, we conclude that the association of VDAC1 causes a large conformational change in the EMC, which substantially reduces the cavity space and modifies the surface electrostatic property of the EMC hydrophilic vestibule.

## DISCUSSION

The EMC complex is involved in multiple biological processes, and one of its well-established functions is to act as an insertase for the insertion of terminal transmembrane helices into the ER membrane [4, 5]. The structures of this complex we reported here in both apo and VDAC1-bound states reveal the intricate and dynamic architecture of this multifunctional transmembrane molecular machine. The apo complex displays a dual-membrane cavity structure (Figure 2C), in which the hydrophilic vestibule, a large cavity that serves as the TMH-binding pocket [23–26], can be fully visualized in our apo structure (Figure 2D). This vestibule exhibits substantial conformational changes as revealed in the VDAC1-bound structure (Figure 5A). It is noteworthy that the association of the EMC with VDAC1 is likely involved in processes different from the classical insertase function of the EMC. Hence, our data expand the understanding of the structural basis and multifunctionality of the EMC complex.

**Figure 6:**
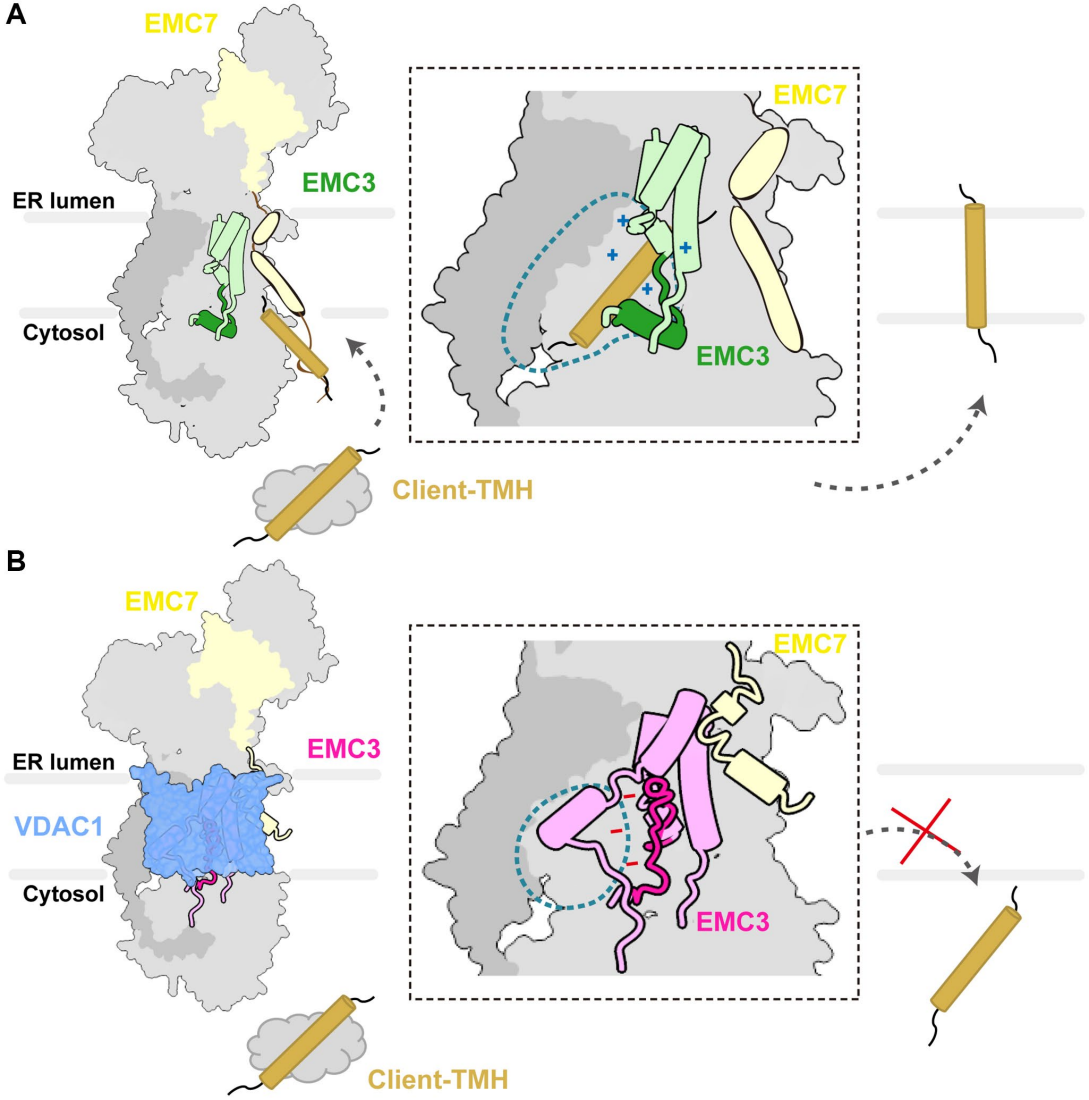
**Schematic diagram of the EMC regulation by VDAC1.** (**A**) The apo conformation of the EMC denotes its role as an insertase primed for binding client TMHs. The zoom-in view shows the hydrophilic vestibule of the EMC complex. The TMH_1–3_ bundle and the L-shape gating plug of EMC3 are shown in light green and green respectively, and client-TMH in brown. The inside of the vestibule is positively charged as indicated. (**B**) The association of VDAC1 induces structural changes in the hydrophilic vestibule. The TMH_1–3_ bundle and the gating plug of EMC3 are shown in dark orchid and orchid, respectively. The hydrophilic vestibule is reduced and the electrostatic surface potential inside is altered to negatively charged.

Based on our structural analysis and previous studies [23–26], we propose a model for EMC regulation. In the apo state, two dynamic elements located near the entrance of the hydrophilic vestibule TMH2 of EMC3 and the TMH and C-terminal cytosolic loop of EMC7 remain idling in the membrane, primed for the binding of substrates or cofactors (Figure 6A). We speculate that these two dynamic elements initially engage and further guide the entrance of substrate-TMHs into the hydrophilic vestibule (Figure 6A). In accordance with this notion, EMC7 deletion mutant that lacks the TMH and C-terminal cytosolic loop and mutations in TMH2 of EMC3 all lead to biogenesis defects of EMC substrates, underscoring important roles of these elements in client insertion (Supplementary Figure 6A) [26]. Within the hydrophilic vestibule, the EMC3^188–210^ gating plug adopts an L-shaped conformation to provide enough space to house the substrate-TMH (Figure 6A). Additionally, a conserved positively charged patch on EMC3 is formed to facilitate substrate binding (Figure 6A), mechanistically similar to bacterial YidC [28]. Consistently, a recent study reported that the EMC3^188–210^ gating plug can directly bind to the substrates [27]. In that work, the EMC3^188–210^ segment was found in a partially open circular conformation, implying its plasticity under varying conditions, including the use of a nanobody to stabilize EMC7 [27]. Taken together, our apo conformation of the EMC denotes its role as an insertase ready for substrate binding.

In the VDAC1-bound state, the EMC undergoes a conformational change to hold tightly to the outer wall of VDAC1 (Figure 6B). It is noteworthy that the two dynamic elements, TMH^EMC7^ and TMH2^EMC3^, substantially contribute to the EMC-VDAC1 interaction (Figure 6B), highlighting their importance in interacting with other factors in addition to client TMHs. Moreover, a salient feature of the EMC-VDAC1 complex is the constriction of the hydrophilic vestibule of the EMC and the alteration of the electrostatic potential inside it, which are mainly induced by the conformational changes of the gating plug in the vestibule (Figure 6B). Recently, the structure of another EMC-client complex, EMC-Ca_v_, was reported [44]. In contrast to the binding of VDAC1 to EMC, Ca_v_ associates with the hydrophobic cavity side of the EMC, suggesting that the EMC employs distinct transmembrane interfaces to interact with different clients. Notably, the EMC3 gating plug in the EMC-Ca_v_ structure forms an open circular loop and occupies the substrate binding cavity of the EMC, which resembles its configuration in the VDAC1-bound state (Supplementary Figure 6B) [44]. We anticipate that the gating plug can prevent simultaneous binding of the EMC to both VDAC1/Ca_v_ and the client TMH, i.e., by ejecting the subtract TMH from the binding pocket prior to the association with VDAC1 or Ca_v_. Collectively, The VDAC1-bound state of the EMC likely represents an additional function of the EMC beyond its primary role in client insertion.

What is the function of the EMC complex in the VDAC1-bound state? VDAC1 biogenesis on the outer mitochondrial membrane is well-established and involves a complex pathway with the Tim/Tom import machinery, sTIM chaperones and the SAM/TOB complex [45]. Previous studies also unveiled that a portion of VDAC1 appears on other membrane systems [46–49], including the ER and plasm membrane [50–52], especially in conditions like type 2 diabetes [46], Alzheimer’s disease [31], and virus infection [53]. However, the mechanism of VDAC1 translocation remains unclear. Recent research has revealed that ER-associated chaperones at mitochondria-ER contact sites, including the EMC, GET and Djp1, can interact with certain mitochondrial membrane protein precursors, aiding in their translocation and sorting [16, 54–56]. Our analysis reveals that the EMC-associated VDAC1 is located at mitochondria-ER contact sites and the EMC-VDAC1 interaction is evolutionarily conserved from yeast to human, implying that it likely occurs under physiological conditions. Intriguingly, there is an increase in the translocation of VDAC1 under certain pathological conditions [46, 52]. We propose that the association between EMC and VDAC1 may potentially strengthen under these abnormal conditions, and the EMC-VDAC1 complex may assist VDAC1 in translocation or carrying out non-classical functions. Future biochemical and functional studies will be required to fully understand the *in vivo* functions of the EMC-VDAC1 complex.

## METHODS

### Cell culture

Human embryonic kidney 293T cells (293T) and Expi293F cells were purchased from the Type Culture Collection of the Chinese Academy of Sciences, Shanghai, China and Thermo Fisher Scientific (A14527, USA), respectively. 293T cells were maintained in high glucose DMEM media supplemented with 10% FBS in 5% CO_2_ at 37°C. Expi293F cells were cultured in Union-293 medium without FBS at 37°C in 5% CO_2_. Plasmid transfection was performed using x-tremeGENE HP transfection reagent (Roche, Switzerland) according to the manufacturer’s recommendations.

### Stable cell line establishment

To prepare human ER membrane protein complex (EMC) for structural study, we established a stable cell line using the lentivirus transfection system to overexpress two subunits of human EMC. EMC5 was cloned into a modified lentivirus expression vector pLVX-IRES-EGFP with a C-terminal twin-strep tag and EMC2 was cloned into a modified lentivirus expression pLVX-IRES-mCherry vector with a C-terminal 3xFlag tag. The constructed lentiviral expression vectors together with the virus packaging plasmids were introduced to 293T cells using x-tremeGENE HP transfection reagent (Roche) according to the manufacturer’s recommendations. After 36 h of transfection, the virus-containing supernatants derived from these 293T cells were used to infect the Expi293F cells. After 12 h of infection, the cells were replaced with fresh medium and three days after infection, the infected cells were subjected to flow cytometry to sort out the EGFP+/mCherry+ double positive cells which indicate the constituent expression of these two target proteins.

### Expression and purification of human EMC complex

6~7 L of Expi293F cells with tagged EMC5 and EMC2 proteins at a density of 4 × 10^6^ cells/mL were harvested by centrifugation at 2,000 rpm for 15 min. The cell pellets were washed once by low salt buffer containing 10 mM HEPES pH 7.5, 20 mM KCl, 10 mM MgCl_2_ and then were harvested by centrifugation at 3,800 rpm. The cell pellets were re-suspended in 270 ml low salt buffer with protease inhibitor cocktails (Roche), 1 mM DTT, 1 mM PMSF and were broken by Dounce homogenizer (Sigma-Aldrich, USA) at 4°C. The crude membranes were washed twice by EMC buffer (50 mM HEPES pH 7.5, 200 mM NaCl, 2 mM Mg (OAc)_2_, 1 mM DTT). The membranes were suspended in solubilization buffer (EMC buffer with 1% (w/v) n-dodecyl-β-D-maltoside (DDM)) with gentle rotation at 4°C for 2.5 h. Insoluble sample was removed by centrifugation at 19,000 rpm for 50 min at 4°C and the supernatant was then applied to Strep-Tactin XT Superflow beads (2-4010-010, IBA, Germany) for overnight binding. The beads were washed with 50 mL 0.1% digitonin EMC buffer. The protein complex was eluted with 0.1% digitonin EMC buffer supplemented with 50 mM biotin. The elution was then applied to anti-DYKDDDDK G1 affinity resin (L00432, GenScript, USA). The gel was then washed extensively and the complex was eluted by 0.1% digitonin EMC buffer supplemented with 200 μg/mL Flag peptides (A6001, ApexBio, USA). The final elution of human EMC complex was concentrated and loaded on to a Superose 6 Increase 5/150GL column. Peak fractions containing the EMC complex were collected and concentrated for biochemical and electron microscopy study.

### Preparation of vitrified sample

A 3-μl droplet of purified sample at a concentration of around 6 mg/ml was applied to glow-discharged holy carbon grids (Quantifoil, 300 mesh copper R1.2/1.3). Excess sample was removed by blotting with filter paper for 3 s before plunge-freezing in liquid ethane using a FEI Vitrobot Mark IV at 100% humidity and 4°C.

### Cryo-EM data acquisition

Images were collected on a FEI Titan Krios microscope at 300 kV using a K3 DDD detector in super-resolution mode at a magnification of 81,000× with a pixel size of 0.55 Å. EPU software [57] was used for automatic data collection. Each stack was exposed for 3.2 s with a dose of 18.906 e^-^ per pixel per second, resulting in a total of 32 frames per stack. The total dose rate was around 50 e^−^/Å^2^ for each stack. Defocus values vary from −1.2 to −2.5 μm.

### Cryo-EM data processing and model building of the human EMC

21,478 movies were processed using Relion-3 [58] to perform drift correction and dose weighting with MotionCor2 [59]. The contrast transfer function (CTF) parameters were estimated by Gctf [60]. An initial subset of approximately 1,000 particles were manually picked and subjected to reference-free 2D classification. The resulting 2D class averages were used as a reference for particle auto-picking using Gautomatch-v0.56 (developed by Kai Zhang). After Gautomatch, 6,138,030 particles were extracted using an optimized box size of 352 pixels. Next, false positives or ‘bad particles’ were eliminated over two rounds of reference-free two-dimensional (2D) classification. A total of 4,194,019 particles were finally selected and subjected to a global angular search 3D classification with 5 classes and 60 iterations. Two classes (28% and 24%) were subjected to 3D auto-refinement, respectively. Then the particles were used for further 3D classification with no alignment, and “good” particles were selected for further auto-refinement. In order to improve the resolution, particles screening was employed. Except for particles max resolution >4 Å, others were subjected to CTF refinement. At last, the particles imported into cryoSPARC [61] non-uniform refinement led to consensus maps at global resolutions of 3.47 Å and 3.32 Å, respectively. All resolutions were based on the gold standard (two halves of data refined independently) Fourier shell correlation (FSC) = 0.143 criterion. [62] For the EMC-VDAC1 complex, a preliminary model was built using the previously reported EMC structure (PDB: 6WW7) [26] and VDAC1 (PDB: 2JK4) [37]. For the Apo-EMC complex, the EMC structure (PDB: 6WW7) [26] was used as starting model. Docked model and maps were further manually checked and fitted in Coot [63], followed by fragment-based refinement with Rosetta [64] and real-space refinement using PHENIX [65]. The models of the complexes were validated by MolProbity. [66] All the structural figures were prepared by using Chimera [67], PyMol (https://pymol.org/). The program HOLLOW was used to analyze the structures of the human EMC [68].

### Knockout hEMC cell lines

To prepare the human EMC7 knockout cell lines for further study, we established a stable cell line using the CRISPR-Cas9 system [69]. A target sequence was cloned into a modified CRISPR-Cas9 vector pX330-mCherry. After that, the constructed vector was introduced to adherent Expi293F cells using x-tremeGENE HP transfection reagent according to the manufacturer’s recommendations. After 48 h infection, mCherry-positive cells were single-cell sorted into 96-well plates using BD FACS Aria II. EMC7 knock-out was confirmed by PCR and Western blots. The adherent Expi293F cells were cultured in DMEM medium supplemented with 10% FBS.

### Flow cytometry analysis

To test the effect of the EMC7 mutation on the EMC function in cells, lentiviral vectors in a pLVX-IRES-Puro were generated which stably integrate and express either wild type or the mutant EMC7 fused with a 3xFlag tag from a CMV promotor-driven open-reading frame. EMC7 WT and EMC7^1–156^ cell lines were generated after lentiviral introduction of the respective proteins into the EMC7 knockout cell line. The cells growing in a 6 cm tissue culture plate were transfected with GFP-2A-RFP SQS^378–410^, GFP-2A-RFP-SOAT1, GFP-2A-RFP-ASGR (150 ng/well), respectively. 24 h after transfection, cells were detached with trypsin/EDTA, pelleted and resuspended in ice-cold PBS, and analyzed by flow cytometry using a FACS Canto (BD Biosciences, USA). 50,000 GFP positive cells were collected and RFP:GFP ratios were determined using FlowJo (v.10). For quantification, mean RFP and GFP fluorescence intensities were determined in GFP-positive cells. RFP: GFP fluorescence ratios were calculated for each sample and normalised to the ratio in WT cells.

### One-step affinity purification of the EMC complex

EMC7 WT and EMC7^1–156^ cells were resuspended in 1% (w/v) DDM (Anatrace, USA) EMC buffer. After incubation on ice for 1 h, the lysate was centrifuged for 20 min at 18,000 × g at 4°C in a table-top centrifuge. The supernatant was then incubated with Flag agarose beads (L00432, GenScript) for 2 h at 4°C. After extensively washing with 0.01% DDM EMC buffer, the complex was eluted by 0.01% DDM EMC buffer supplemented with 200 μg/mL Flag peptides (A6001, ApexBio). Final elution sample was subjected to silver stained SDS-PAGE, Western blot and EM analyses.

### NanoBiT assay

NanoLuc Binary Technology is a protein complementation assay composed of a large part (17.6 kDa or Lgbit) and a small part (11 amino acids or smbit), that can be used for intracellular detection of protein:protein interactions. [69] cDNA fragments of human EMC7, VDAC1, GST, EMC7^1–156^, EMC4 were constructed into PRK and fused at their C termini with either Lgbit or smbit. As a result, EMC7-smbit, GST-smbit, EMC7^1–156^-smbit, EMC4-smbit and VDAC1-Lgbit were constructed. Cells were seeded in 96-well plates at a density of 2.5 × 10^4^ cells per well 6 h prior to transfection. A mixture containing 50 ng of the fused Lgbit plasmid and 50 ng fused smbit plasmid were prepared and added to each well. After 24 h of transfection, the substrate (furimazine) was added, and the cells were subjected to detect the luminescence.

### Bimolecular fluorescence complementation assay

Bimolecular Fluorescence Complementation (BiFC) assay is a method used to directly visualize protein-protein interaction *in vivo* using live-cell imaging or fixed cells [39, 40]. cDNA fragments of human EMC2, EMC5, EMC7 and VDAC1 were constructed into pcDNA3.4 and fused at their C termini with a Venus protein (super-enhanced YFP) N-terminus (1–154) (-VN) or C-terminus (155–238) (-VC). Thus, EMC5-VN, EMC2-VC, EMC7-VC, VDAC1-VN were constructed. EMC5-VN and EMC2-VC were co-transfected into 293T cells with the x-tremeGENE HP transfection reagent according to the manual, and so do EMC7-VC and VDAC1-VN. After 24 h, the 293T cells were subjected to STED analysis for Venus signals.

### SMALP formation and protein purification

EMC7-His and EMC5-Strep Expi293F cells were treated with 0.5 mM EGS (ethylene glycol bis) for 30 min under gentle nutation at RT. The cells were then washed with membrane buffer containing 50 mM Tris-HCl (pH 8.0) and 400 mM NaCl, and harvested by centrifugation at 2000 rpm. The cell pellets were resuspended in membrane buffer with protease inhibitor cocktails. The suspended cells were frozen with liquid nitrogen and crushed into 35 mm beads. The frozen cell beads were further disrupted using SPEX 6870D Freezer Mill. The lysate was centrifuged at 3900 rpm for 30 min at 4°C. The supernatant was collected and subjected to an additional centrifugation at 100,000 g for 60 min at 4°C. The membrane pellet was collected and then resuspended in membrane buffer and solubilized for 2 h at 4°C with 2% (wt/vol) SMA (styrene maleic acid) copolymer (~3:1 M ratio of styrene:maleic acid, Cray Valley, USA). After incubation, the mixture was centrifuged for 60 min at 100,000 g to remove insolubilized membrane. The supernatant was mixed with 5 mL Ni-Sepharose resin (GE Healthcare, USA) and incubated for 12 h under gentle nutation at 4°C. The resin was washed with 30 CV membrane buffer supplemented with 30 mM imidazole and eluted with 5 CV membrane buffer supplemented with 250 mM imidazole. The eluate was applied to Strep-Tactin XT Superflow beads (IBA, 2-4010-010) with gentle rotation at 4°C for 6 h. The beads were washed with 50 mL membrane buffer. Finally, the protein complex was eluted using membrane buffer with 50 mM biotin and concentrated for further biochemical studies.

### Yeast strains

The *S. cerevisiae* BCY123 strains were used for the purification. A TAP-tag was introduced at the C-terminus of BCY123 strains’ EMC7 with a Hygromycin B selection marker, following a 3xFlag tag introduced at the C-terminus of VDAC1 with a KanMX4 selection marker [70]. All the genetic manipulations in yeast were followed by standard lithium acetate transformation method [71]. All the achieved strains had been confirmed by PCR-sequencing and Western blots.

### Purification of the endogenous yeast EMC complex

Yeast cells were cultured in YPAD medium for ~ 24 h at 30°C to an OD600 of 3~5, and harvested by centrifugation at 3,500 rpm. The cell pellets were washed and then resuspended in EMC buffer containing 50 mM HEPES pH7.5, 200 mM NaCl, 2 mM Mg (OAc)_2_, 1 mM DTT and protease inhibitor cocktails. The suspended cells are frozen to 35 mm beads by liquid nitrogen. The frozen cell beads were broken down by SPEX 6870D Freezer Mill. Lysate was centrifuged at 10,000 g for 30 min at 4°C. The supernatant was collected and centrifuged at 100,000 g for 60 min at 4°C. The membrane pellet was collected and then resuspended in solubilization buffer (EMC buffer with 1% (w/v) n-dodecyl-β-D-maltoside (DDM) with constant rotation at 4°C for 2.5 h. After incubation, the mixture was centrifuged for 30 min at 120,000 g to remove insolubilized membrane. The supernatant was mixed and incubated with the IgG-coupled magnetic beads and cleaved by TEV protease overnight in the 0.01% DDM EMC buffer. The eluate was then incubated with Flag agarose beads (GenScript Biotech, L00432) for 2 h at 4°C. After extensively washing with 0.01% DDM EMC buffer, the complex was eluted by 0.01% DDM EMC buffer supplemented with 200 μg/mL Flag peptides (ApexBio, A6001). Final elution sample was detected by SDS-PAGE and mass spectrometry.

### Mass spectrometry analysis

The purified complexes were reduced with 10 mM Tris (2-carboxyethyl) phosphine hydrochloride (TCEP) and alkylated with 25 mM Iodoacetamide. After protein precipitation by chilled acetone, the proteins were reconstituted in 25 mM ammonium bicarbonate buffer and digested with sequencing grade trypsin (Promega) in an enzyme/substrate ratio of 1:50 at 37°C overnight. For liquid chromatography tandem mass spectrometry (LC-MS/MS) analysis, the digested peptide mixtures were loaded onto a trap column (2 cm × 100 μm inner diameter (i.d.), C18, 3 μm particle size, 100 Å pore size) for online desalting using the EASY-nLC 1200 UHPLC (Thermo Fisher Scientific). Peptides were then separated on an in-house packed column (15 cm × 75 μm i.d., ReproSil-Pur C18, 1.9 μm particle size, 120 Å pore size, Dr. Maisch) at a flow rate of 300 nL/min. The eluate was directly introduced into an Orbitrap Fusion mass spectrometer or Q Exactive HF mass spectrometer (Thermo Fisher Scientific). Mass spectra were acquired in a data-dependent mode, each full scan MS (m/z 350–1500, resolution 60 K) was followed by several higher collision-induced dissociation (HCD) MS/MS scans for the most intense precursor ions. The raw data were extracted and searched by Proteome Discoverer 2.2 (Thermo Fisher Scientific) with the SEQUEST search engine against the human and yeast UniProt database. Enzyme specificity was set to trypsin, allowing for up to 2 missed cleavages. Mass tolerance was set to 10 ppm for the precursor ions and 0.02 Da for fragment ions. The carbamidomethylation (+57.022 Da) of cysteine was set as static modifications, and oxidation of methionine residues (+15.995 Da) was set as a variable modification. The decoy database searches were also performed in parallel, and peptides less than 1% false discovery rate (FDR) were accepted.

## Supplementary Materials

Supplementary Figures

Supplementary Table 1
