# Supplementary material for: Structural insights into human EMC and its interaction with VDAC
Source: Aging (Albany NY). 2024 Mar 15;16(6):5501–25. doi: 10.18632/aging.205660 (PMC11006472; doi:10.18632/aging.205660)
Supplement: Supplementary Figures [file aging-16-205660-s001.pdf]

SUPPLEMENTARY FIGURES

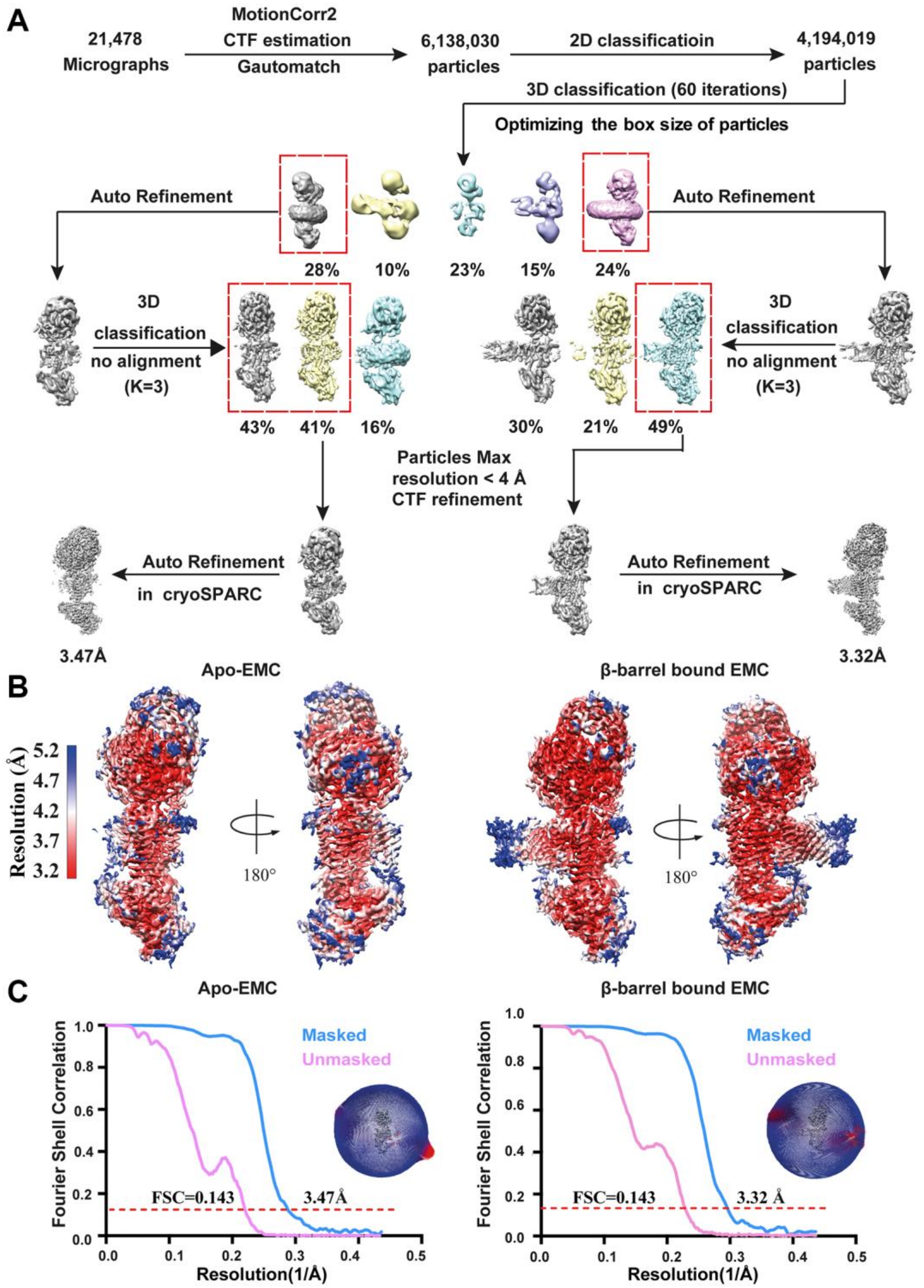

Supplementary Figure 1. Cryo-EM data processing and structure determination of human EMC complexes. (**A**) Data processing of single particle images of human EMC leading to high-resolution reconstructions of the complexes in both apo and VDAC-bound states. (**B**) Final 3D density maps colored according to the local resolution. (**C**) Gold-standard Fourier Shell Correlation (FSC) curves of the overall map and angular distribution of the particles used for the final reconstruction. All the images in this figure are created by Chimera X.

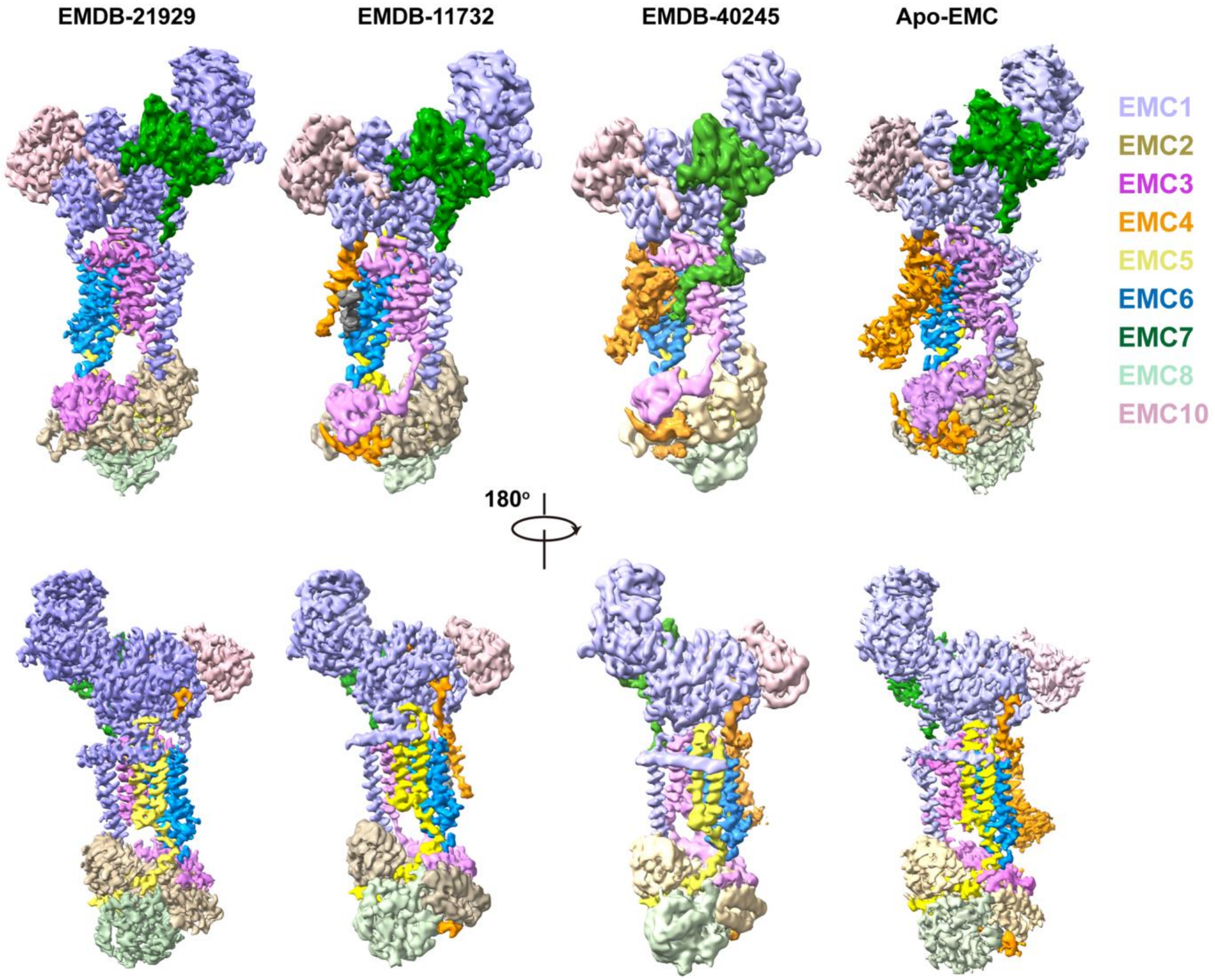

Supplementary Figure 2. Structural comparison of the human EMC complex reported here with those from previous studies. The published human EMC density maps, including emdb-21929 at 3.4 Å, emdb-11732 at 3.4 Å, and emdb-40245 at 3.5 Å as well as our apo EMC density map at 3.47 Å, are used for the comparison. The EMC subunits are color-coded.

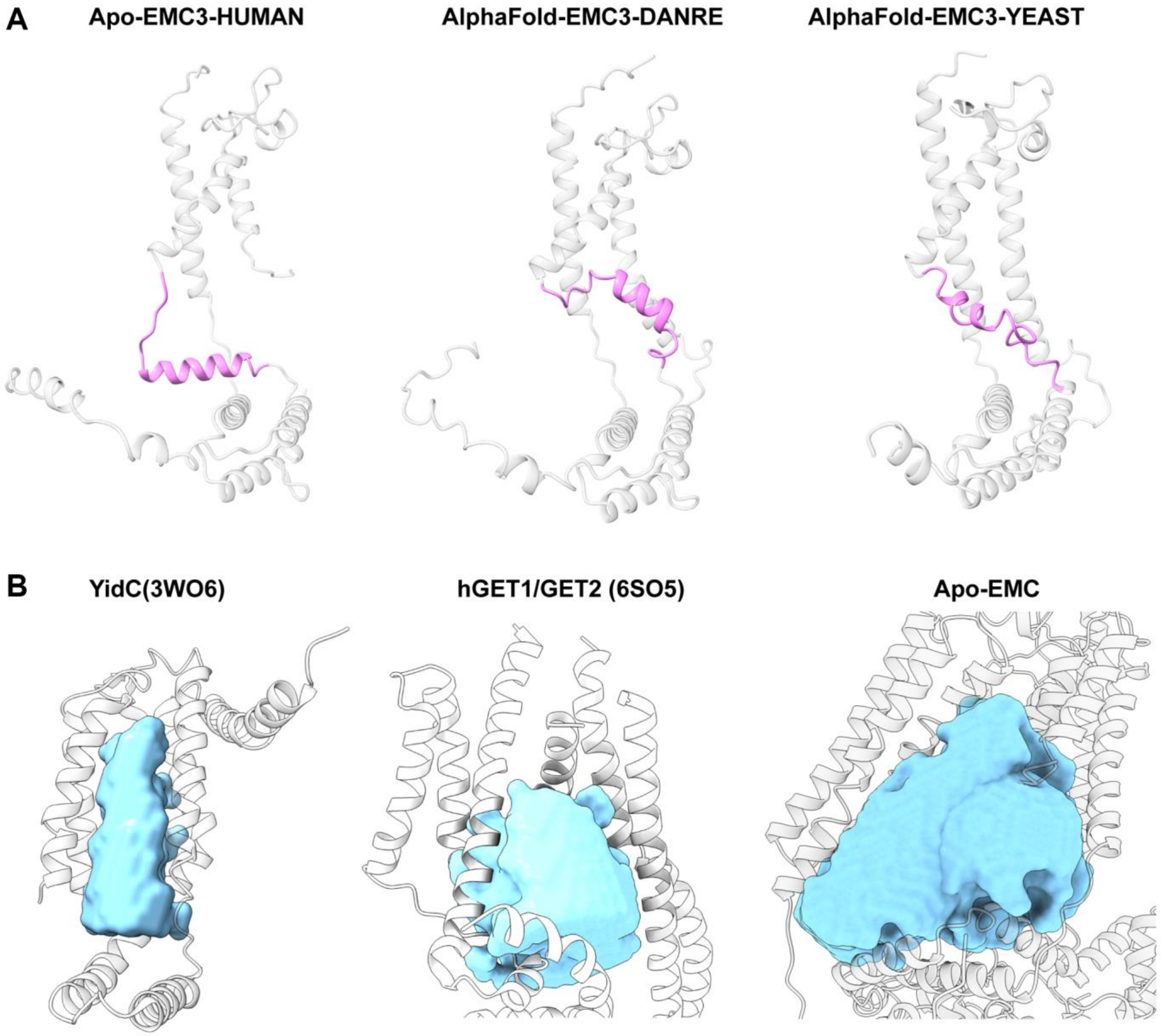

Supplementary Figure 3. The 'loop-helix' structural pattern of the EMC3$^{188–210}$ segment and comparison of the hydrophilic insertase pocket in the Oxa1 superfamily members. (**A**) Structural comparison of the human EMC3$^{188–210}$ segment with its orthologs in yeast (AF-P36039-F1, predicted by Alpha-fold) and zebrafish (AF-Q7SXW4-F1, predicted by Alpha-fold) shows a conserved 'loop-helix' structural pattern. (**B**) The hydrophilic cavities in *Bacillus halodurans* YidC (PDB: 3WO6), human GET1/GET2 (PDB: 6SO5) and human EMC (this study), as determined by program HOLLOW, are shown as surface representation. Proteins are shown in the cartoon model and colored in gray.

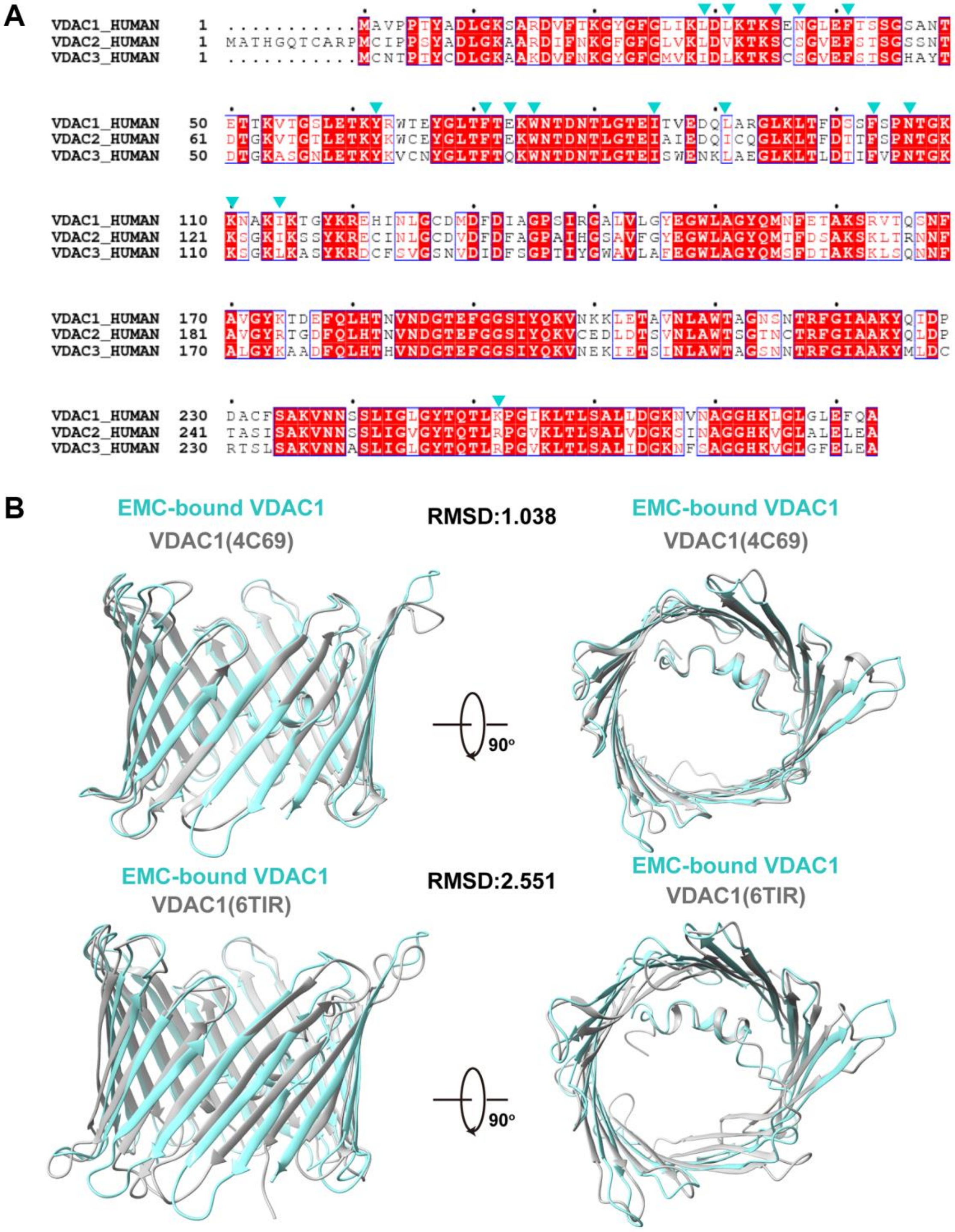

Supplementary Figure 4. Sequence alignment analysis of human VDAC paralogs and structural comparison of VDAC1 in different states. (**A**) The three human VDAC paralogs, VDAC1 (P21796), VDAC2 (P45880) and VDAC3 (Q9Y277), share a high sequence homology (65% to 70% identity, 80% to 90% overall similarity). The majority of the interface residues on VDAC1 (cyan arrowheads) revealed by the EMC- VDAC1 complex structure, are conserved in both VDAC2 and VDAC3. (**B**) The EMC-bound VDAC1 (this study) is respectively superimposed with the open state VDAC1 (ATP-bound, PDB: 4C69) and the closed state VDAC1 (NADH-bound, PDB: 6TIR)

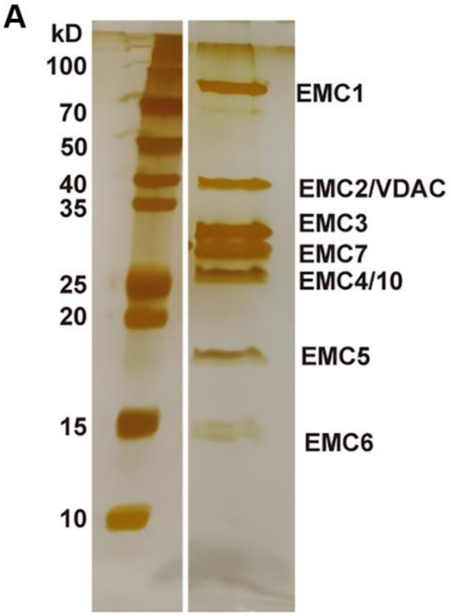

B **Mass Spectrometric Analysis of Yeast EMC**

Uniprot Acc. No.	*S.cerevisiae* protein	PSM	Unique peptides
P25574	EMC1	330	54
P04840	VDAC1	178	20
P47133	EMC2	134	22
Q12025	EMC10	99	13
P36039	EMC3	72	12
P53073	EMC4	65	23
P39543	EMC7	60	15
P40540	EMC5	33	12

Supplementary Figure 5. Purification of *Saccharomyces cerevisiae* (*S.c*) EMC complex. (**A**) A silver staining image of the SDS-PAGE gel for the purified *Saccharomyces cerevisiae* (*S.c*) EMC complex. (**B**) Mass spectrometry analysis of the purified yeast EMC.

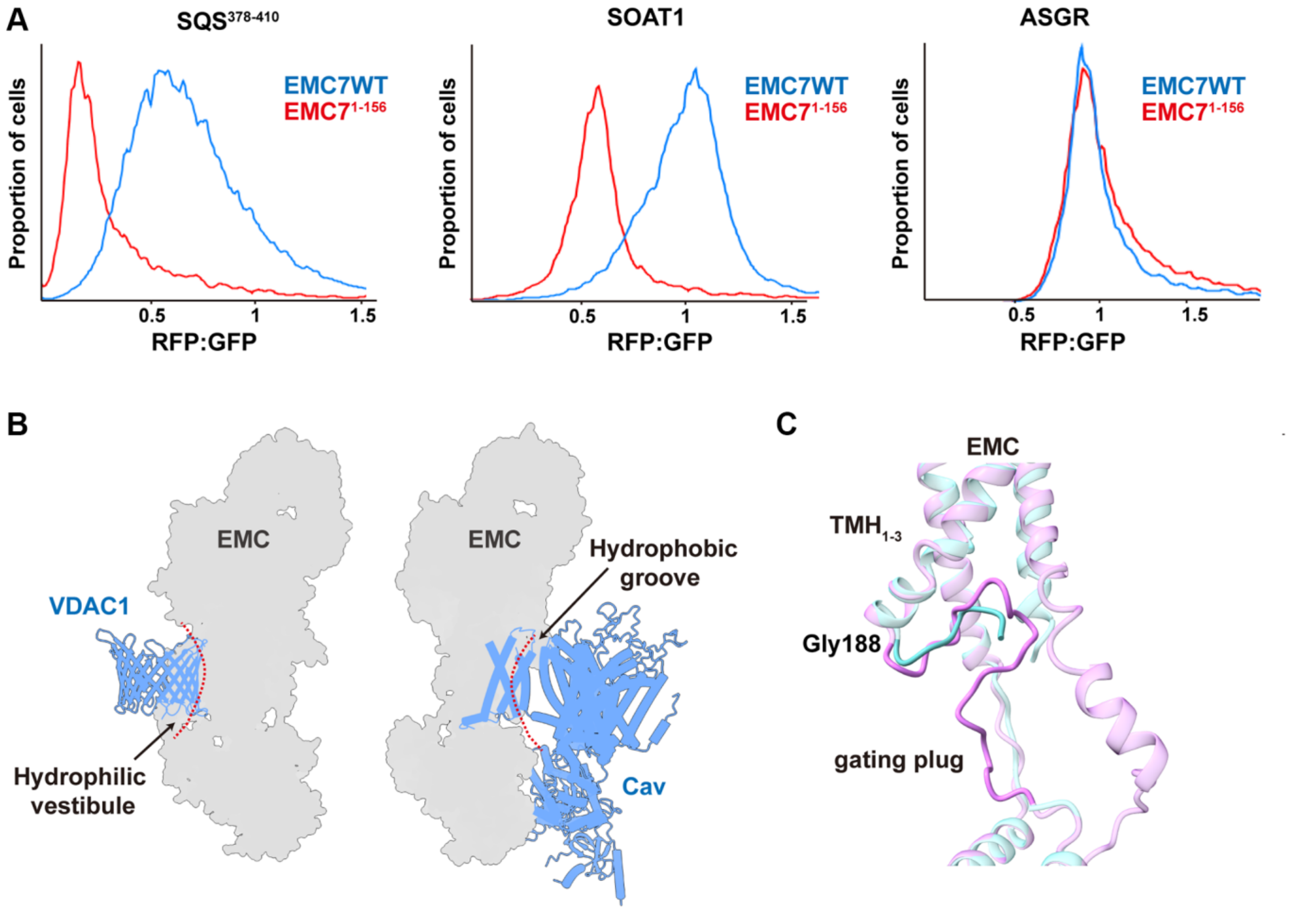

Supplementary Figure 6. The TMH and C-terminal cytosolic loop of EMC7 is required in client insertion and structural comparison of the EMC-VDAC1 and EMC-Cav complexes. (**A**) Fluorescent client reporter stability assay was performed with Expi293 cells stably expressing exogenous WT EMC7 or the truncation mutant EMC7$^{1–156}$ that lacks the TMH and C-terminal cytosolic loop, and transiently expressing the substrates GFP-T2A-RFP-SQS$^{378–410}$ (EMC-dependent), GFP-T2A-RFP-SOAT1 (EMC-dependent) or GFP-T2A-RFP-ASGR (EMC-independent). The relative RFP fluorescence signals are normalized to the internal expression control (GFP). (**B**) Schematic diagrams of the interfaces between EMC-VDAC1 and EMC-Cav. (**C**) Conformational change of EMC3$^{188–210}$ segment, the gating plug, between the VDAC1-bound (orchid) and Cav-bound states (cyan). This segment forms an open circular loop in both the VDAC1-bound and Cav-bound states, held against the TMH1-3 bundle of EMC3.
