# Supplementary material for: Structural insights into human EMC and its interaction with VDAC
Source: Aging (Albany NY). 2024 Mar 15;16(6):5501–25. doi: 10.18632/aging.205660 (PMC11006472; doi:10.18632/aging.205660)
Supplement: Supplementary Table 1 [file aging-16-205660-s002.pdf]

SUPPLEMENTARY TABLE

Supplementary Table 1. Cryo-EM data collection and refinement statistics.

	Apo-EMC<br>EMD-35906<br>PDB 8J0N	EMC-VDAC1<br>EMD-35907<br>PDB 8J0O
**Data collection and processing**		
EM equipment	FEI Titan Krios	FEI Titan Krios
Magnification	81,000	81,000
Voltage (kV)	300	300
Detector	Gatan K3	Gatan K3
Pixel size (Å)	1.09	1.09
Electron dose (e-/Å^2)	50	50
Defocus range (μm)	–1.2 to –2.5	–1.2 to –2.5
**Reconstruction**		
Software	Relion 3.0, cryoSPARC	Relion 3.0, cryoSPARC
Number of used particles	693,381	455,504
Final resolution (Å)	3.47	3.32
FSC threshold	0.143	0.143
Map sharpening B-factor (Å^2)	–120.0	–122.9
**Model building**		
Software	Coot 0.89	Coot 0.89
**Refinement**		
Software	Phenix 1.20.1	Phenix 1.20.1
Map CC	0.70	0.72
**Model composition**		
Protein residues	2,301	2,627
Protein atoms	18,475	20,986
**Validation**		
R.M.S deviations		
Bond lengths (Å)	0.010	0.014
Bond angles (°)	0.948	1.083
Ramachandran plot (%)		
Preferred region	91.50	91.37
Allowed region	8.23	8.36
Outlier region	0.26	0.27
MolProbity score	2.01	2.13
Clashscore (all-atom)	9.72	12.97
Poor rotamers (%)	0.75	0.40
